# CLCNt2 Mediates Nitrate Content in Tobacco Leaf, Impacting the Production of Tobacco-Specific Nitrosamines in Cured Leaves

**DOI:** 10.3389/fpls.2022.741078

**Published:** 2022-02-16

**Authors:** Lucien Bovet, Prisca Campanoni, Jian Lu, Aurore Hilfiker, Samuel Kleinhans, Hélène Laparra, Joanne Schwaar, Ramsey S. Lewis, Yuki Matsuba, Hong Ma, Ralph E. Dewey, Simon Goepfert

**Affiliations:** ^1^PMI R&D, Philip Morris Products S.A., Neuchatel, Switzerland; ^2^Department of Crop and Soil Sciences, North Carolina State University, Raleigh, NC, United States

**Keywords:** CLCNt2, chloride channel, tobacco, NNN, NNK, nitrate transport, chloride/proton antiporters, tobacco-specific nitrosamines

## Abstract

Nitrate accumulation in tobacco (*Nicotiana tabacum* L.) leaf, particularly in the burley (BU) type, is a reservoir for the generation of nitrosating agents responsible for the formation of tobacco-specific nitrosamines (TSNAs). TSNAs are mainly produced *via* the nitrosation of alkaloids occurring during the curing of tobacco leaves. Additional formation of TSNAs may also occur during tobacco storage, leaf processing and in some circumstances *via* pyrosynthesis during combustion. Two TSNA species, 4-(methylnitrosamino)-1-(3-pyridyl)-1-butanone (NNK) and N-nitrosonornicotine (NNN) are found in the tobacco products and have been documented to be animal carcinogens. A previous study showed that decreasing the accumulation of nitrate in tobacco leaf *via* the overexpression of a deregulated form of nitrate reductase is efficient to reduce the production of TSNAs. We pursue in finding another molecular genetic target to lower nitrate in BU tobacco. Suppressing expression or knocking-out *CLCNt2* has a direct impact on leaf nitrate and TSNA reduction in cured leaves without altering biomass. This study provides now a straight path toward the development of new commercial tobacco varieties with reduced TSNA levels by breeding of variants deficient in active *CLCNt2* copies.

## Introduction

The International Agency for Research on Cancer has identified eight tobacco-specific nitrosamines (TSNAs) in tobacco and tobacco smoke (IARC, 2004 and the references therein). They are unique to tobacco and are present in smokeless tobacco, snuff, cigarettes, and electronic cigarette liquids. Two TSNAs have been classified as group 1 carcinogens: *N*-nitrosonornicotine (NNN) and 4-methyl-*N*-nitrosamino-1-(3-pyridyl)-1-butanone (NNK). For this reason, strategies for reducing TSNA levels in tobacco are being developed. To date, no genetic solution has been found to significantly reduce the levels of NNK. In contrast, a technique known as the Zyvert™ technology (Lusso et al., 2017) can reduce the levels of NNN and, specifically, its precursor nornicotine by knocking out three genes that encode the cytochrome P450 nicotine demethylases that convert nicotine to nornicotine (Lewis et al., 2010).

**Figure 1:**
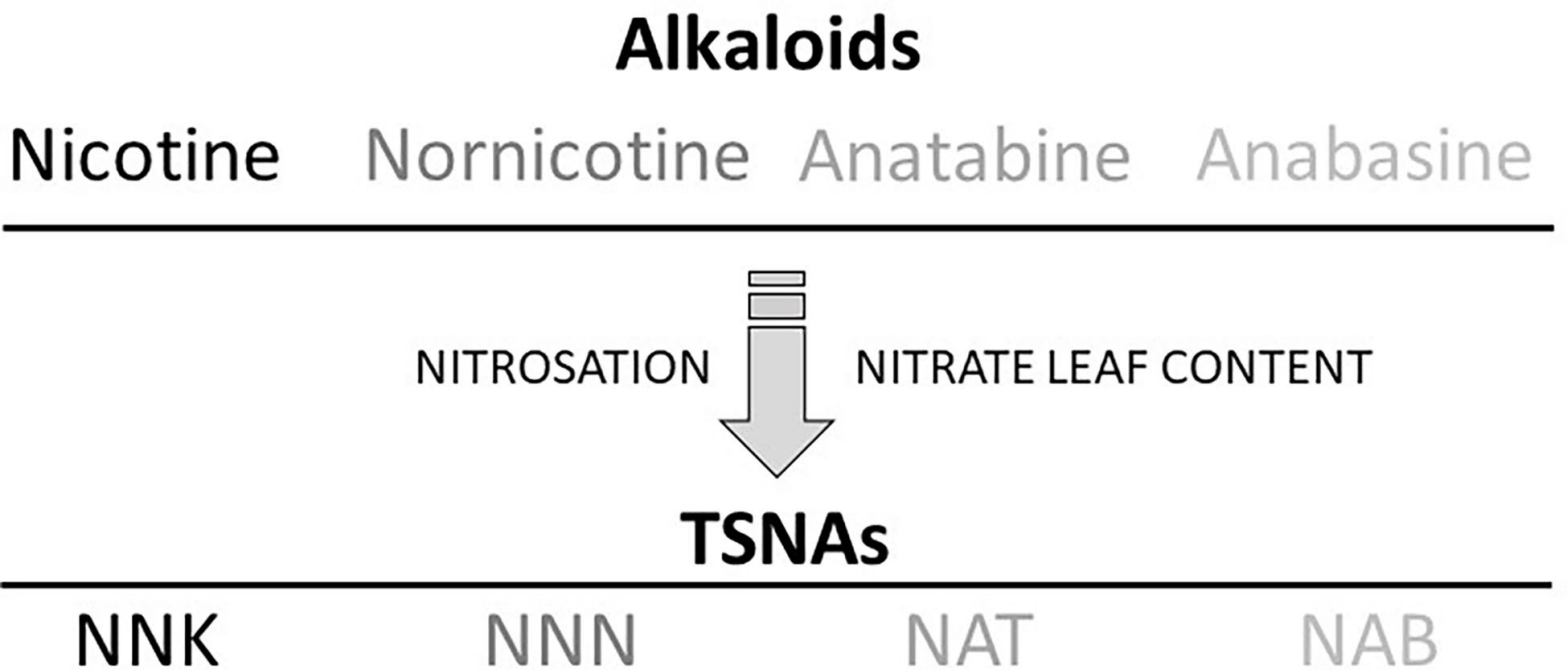
Tobacco-specific nitrosamine (TSNA) formation in tobacco. Nitrosating agents essentially derived from nitrate storage pools react with the tobacco alkaloids nicotine, nornicotine, anatabine, and anabasine to generate the respective TSNAs, 4-(methylnitrosamino)-1-(3-pyridyl)-1-butanone (NNK), *N*’-nitrosonornicotine (NNN), *N*-nitrosoanatabine (NAT), and *N*-nitrosoanabasine (NAB).

Tobacco-specific nitrosamines are generated in tobacco through the nitrosation of alkaloids. The principal TSNA molecules found in tobacco products—NNK, NNN, *N*-nitrosoanatabine (NAT), and *N*-nitrosoanabasine (NAB)—are directly produced during the curing process through a reaction of nitrite or any other nitrogen oxide (NOx) species with nicotine (or its oxidized derivative pseudooxynicotine; Hu et al., 2015), nornicotine, anatabine, and anabasine, respectively (Burton et al., 1989; Staaf et al., 2005; Figure 1). Curing is an agronomic practice used for tobacco leaf maturation and drying (Kentucky Cooperative Extension, 2015). Bacterial populations present on the tobacco leaf surface are suspected to propagate during curing, thereby facilitating the reduction of nitrate to nitrite or other NOx species to catalyze the nitrosation of the secondary amines in alkaloids (Wiernik et al., 1995; Rundlöf et al., 2000). Post-curing storage of tobacco under improper conditions may also contribute to increased TSNA levels as the result of active nitrosation (Shi et al., 2013).

Plant genetics, agronomic practices, and curing conditions all contribute to the accumulation of TSNAs across the different tobacco types. The burley (BU) market type is known to contain higher TSNA concentrations than the two other main commercial types, namely Virginia [also referred to as flue-cured (FC) tobacco] and oriental (OR; Burton et al., 1994; Li et al., 2017a). Interestingly, BU tobaccos also require exceptionally high nitrogen fertilization rates and accumulate more nitrate within the leaf (Li et al., 2017b). Therefore, BU tobaccos also assimilate greater amounts of nitrogen into amino acids and proteins compared to Virginia or OR types (Bovet et al., 2020). A substantial portion of the soil nitrate transported from root to shoot is reduced to ammonia in the chloroplast and assimilated into amino acids, serving as the major nitrogen source for the plant (Lam et al., 1996, Coruzzi, 2003). It is the pool of unassimilated nitrate stored as such in the leaf vacuole; however, that appears to play the greatest role in the bacteria-mediated conversion to nitrite (or other NOx species) during curing, contributing to the nitrosation reactions that promote the active formation of TSNAs (Law et al., 2016; Wang et al., 2017). We have shown previously that a dramatic reduction in the nitrate content in BU leaves can be realized through constitutive expression of a nitrate reductase enzyme that can no longer be downregulated *via* reversible phosphorylation, this constitutive expression is achieved through substitution of an Asp residue for Ser523 (Lu et al., 2016, 2021). The low-nitrate phenotype in these plants is accompanied by a substantial reduction in the TSNA content of the cured leaf. These studies highlight the importance of nitrate storage pools in the leaf in driving TSNA formation during tobacco air-curing.

Under conditions of excess nitrate availability, plants store this critical nutrient in the vacuole and, on demand, release it into the cytoplasm (Shitan and Yazaki, 2013). In *Arabidopsis*, AtCLCa—a member of the chloride channel (CLC) protein family, which includes both chloride (Cl^−^) channels and chloride/proton (Cl^−^/H^+^) antiporters—mediates nitrate accumulation into the vacuole. Interestingly, AtCLCa knockout (KO) plants show a nitrate underaccumulation phenotype at the whole-plant level (Geelen et al., 2000; De Angeli et al., 2006; Monachello et al., 2009).

In this study, we identified putative tobacco orthologs of AtCLCa and AtCLCb (a close relative of AtCLCa also involved in nitrate transport into the vacuole in *Arabidopsis*; von der Fecht-Bartenbach et al., 2010). *AtCLCa*–*b* tobacco orthologs are part of the 17 CLC genes identified in the genome of *Nicotiana tabacum* (Zhang et al., 2018). These genes, originating from the two tobacco ancestors *Nicotiana sylvestris* and *Nicotiana tomentosiformis* (Sierro et al., 2013), were named *CLCNt2-S* and *CLCNt2-T*, respectively, according to a previous work published by Barbier-Brygoo et al. (2000). The purpose of this study was: (i) to determine the degree of nitrate reduction in BU plants subjected to suppressed expression using a RNAi approach and to consolidate the results *via* the knockout of *CLCNt2* using CRISPR–Cas9 technology and (ii) to assess its impact on TSNA reduction in cured leaves and cigarette smoke.

## Materials and Methods

### Plasmid Constructs and Plant Materials

The specific DNA fragment selected for suppressing the expression of both copies of *CLCNt2* (*CLCNt2-S* and *CLCNt2-T*) spans the exon–intron borders between coding exons 4 and 5: 5′-gtcatcatcaggtgtgtgtgctgctttccgttctccagtaggtggtgtcctatttgctttagaggaagtggcaacatggtggagaagtgcactcctctggagaactttcttcagcacggcagttgtggtggtgatactgagggccttcattgaatactgcaaatctggcaactgtggactttttggaagaggagggcttatcatgtttgatgtgagtggtgtcagtgttagctaccatgttgtggacatcatccctgttgtagtgattggaatcataggcggacttttgggaagcctctacaatcatgtcctccacaaaattctgaggctctacaatctgatcaacgagaagggaaaactacataaggttcttctcgctctgagtgtctcccttttcacctccattt-3′.

This insert was cloned in both sense and antisense orientations (separated by an intron) between the strong constitutive Mirabilis Mosaic Virus (MMV) promoter and the 3′ nos terminator sequence of the nopaline synthase gene of *Agrobacterium tumefaciens* (Cheng et al., 1997) within a PMI-proprietary binary vector (WO2012098111).

For the suppressed expression, the BU tobacco variety TN90e4e5 was transformed using standard *Agrobacterium*-mediated transformation protocols (Horsch et al., 1985). TN90e4e5 represents a selection from an ethylmethane sulfonate-mutagenized BU population that contains KO mutations in CYP82E4 and CYP82E5v2 (Lewis et al., 2010) to prevent nornicotine production and, therefore, NNN accumulation. Minimizing the nornicotine content in tobacco is highly desirable, because this compound serves as the direct precursor in the synthesis of NNN. Nornicotine is likely produced almost entirely *via* the N-demethylation of nicotine, in a process called nicotine conversion that is catalyzed by the enzyme nicotine N-demethylase (NND). Previous studies have identified CYP82E4 as the specific NND gene responsible for the unstable conversion phenomenon, and CYP82E5v2 as a putative minor NND gene (Lewis et al., 2010). Therefore, we used TN90e4e5 as background to silence the expression of *CLCNt2* to confirm specifically the impact of potential reduced nitrate on the accumulation of all TSNAs, particularly NNN which can be also generated in part by the accumulation of nornicotine. Ten independent T0 plants from each construct were assayed by quantitative reverse transcription PCR (qRT-PCR) to select transgenic lines. T1 seeds were harvested from three independent T0 plants that exhibited the strongest *CLCNt2* silencing. T1 plants were grown in the greenhouse and selected for the presence of construct insertion in the genomic DNA by PCR with the following primers (5′–3′): MMV-F (gacgtctaatcccaacttcgtc) and IPMS2-R (gacgtctaatcccaacttcgtc).

### Phylogenetic Tree

A multiple sequence alignment was generated using Clustal Omega (version 1.2.4, with the options --full--full-iter parameters), and a phylogenetic tree was constructed from these multiple sequence alignments by using FastTree (version 2.1.10, with the options-gamma-pseudo-spr4-mlacc2-slownni). The resulting phylogenetic tree was re-rooted on the longest branch and ordered on the leaf labels, while preserving the tree topology by using the nw_re-root and nw_order programs from newick_utils (version 1.6). The phylogenetic tree was visualized by using ete3 (version 3.1.1). Labels on the nodes are local support values calculated with the Shimodaira–Hasegawa test. The sequences mentioned are available at https://www.solgenomics.net/organism/Nicotiana_tabacum/genome.

### *CLCNt2* Genomic PCR

Specific primers were designed to amplify DNA fragments by using genomic DNA: *CLCNt2*-S (forward primer: aaaacatggctggttacaaacttc; reverse primer: tctaaagcaaataggacaccaccta) and *CLCNt2*-T (forward primer: agtgacagcctcccttatcaatct; reverse primer: cacttcctctaaagcaaataggaca). As internal control (house-keeping gene), tubulin (TUB) transcripts were amplified using forward primer atttgttgactggtgcccaac and reverse primer tcttcatcgtcaacttcagca. PCR amplification of genomic *TUB*, *CLCNt2-S*, and *CLCNt2-T* fragments was performed by using leaf DNA as matrix template isolated from leaves of the three main *N. tabacum* types BU (TN90), FC (K326), and OR (Basma Xanthi) and from the tobacco ancestors *N. sylvestris* and *N. tomentosiformis* cultivated in the greenhouse using DNeasy Plant Mini Kit (Qiagen, Hilden, Germany).

### RNA-Seq Analyses With Field Grown BU TN90

Burley variety TN90 plants were grown in a Swiss field. After flowering, the petal, sepal, immature flower, stem, root, and upper, middle, and lower leaves were collected in triplicate and immediately frozen in liquid nitrogen. Total RNA was isolated from each tissue sample (*n* = 3) by using the RNeasy mini kit (Qiagen, Hilden, Germany). Before cDNA library construction, RNA purity and integrity were checked using the Nanodrop device (Qiagen, Hilden, Germany). mRNA was enriched from total RNA by using a polyA column to generate cDNA libraries for sequencing. All libraries were sequenced on an Illumina HiSeq-2500 sequencer (Illumina, San Diego, CA, United States) by using version 3 chemistry and flow cells with runs of 2 × 100 bases. The reads were cleaned using Trimmomatic (version .32; Bolger et al., 2014) and aligned to the genome using Hisat2 (version 2.0.1 beta; Kim et al., 2015). Cuffdiff2 (version 2.2.1; Trapnell et al., 2013) was used for calculating the FPKM values based on the aligned reads. The transcriptomes were derived by mapping RNA-Seq reads from each tissue to the following tobacco reference genome.^1^ For each plant, three biological replicates were obtained for each tissue. Previously published gene models were used as the basis for differential gene expression analysis (Sierro et al., 2014).

### qRT-PCR Analysis

The effectiveness of the RNAi constructs in silencing *CLCNt2* expression was assessed by qRT-PCR in a greenhouse environment. RNA was isolated from transgenic plants from each independent transformation event and their corresponding control plants, and qRT-PCR was performed using primers (5′ to 3′) F1 (tcccaacatgtacggagcaa) and R1 (aactgctgcaatgcttccaa) and an internal probe (acactttttgtcaagatc). To measure isoform-specific expression of the two *CLCNt2* isoforms in different tissues of the greenhouse-grown TN90 plants, the following primer pairs were used: Nt2S-F (cttttgggaagcctctacaatca) and Nt2S-R (agtaccaggacaagacccgg) for *CLCNt2-S*; and Nt2T-F (cttttgggaagcctctacaattg) and Nt2T-R (agtgccaggacaagaccctt) for *CLCNt2-T*. The qRT-PCR reactions which were normalized against a tobacco actin gene by using the following primers: NtACT-F (cctcacagaagctcctcttaatc) and NtACT-R (acagcctgaatggcgatatac). Each PCR well contained .01 μM of forward and reverse primers, 2 μl of cDNA, and 5 μl of iTaq Universal SYBR Green Supermix (Bio-rad) per a 10-μl reaction mix. The reaction mix was heated to 95°C for 3 min and then amplified over 40 cycles of 95°C for 5 s and 62°C for 15 s.

### Field Design

All field evaluations were conducted at Clayton (NC, United States) using a randomized complete block design with individual plants as the experimental units (39–50 plants per genotype). Agronomic practices typical for BU tobacco production, including topping, were employed. The plants grew for 95 ± 9 days from transplant to harvest time, after which they were cured for 56 ± 10 days. For field-grown samples, leaf tissues were analyzed for nitrate content at the North Carolina State University Tobacco Analytical Service Laboratory as previously described (Lu et al., 2021).

### Alkaloid and TSNA Analysis of Air-Cured Leaves

Nicotine, nornicotine, anatabine, and anabasine levels in cured upper-stalk leaf samples were quantitated using a Perkin–Elmer AutosystemXL Gas Chromatograph in accordance with previously established protocols (Jack et al., 2007). Total alkaloid content was calculated as the sum of nicotine, nornicotine, anatabine, and anabasine levels. NNN, NNK, NAT, and NAB were quantified in accordance with “Method 1” of Morgan et al. (2004). Total TSNA levels represent the sum of NNN, NNK, NAT, and NAB levels.

### Cigarette Smoke TSNA Analysis

Test cigarettes were handmade and filled with the electric cigarette injector “Powermatic 2”^2^ by using standard cigarette tubes. The weight of the cut filler was varied (average, 800 mg) to obtain a resistance-to-draw corresponding to 120–140 mm water gauge. The cigarettes were subjected to machine smoking as outlined by Health Canada Method T-115 (1999). Mainstream smoke was collected on Cambridge filter pads and extracted with 100 mM ammonium acetate containing D4-NNN and D4-NNK standards. The extracts were analyzed by LC–MS/MS, liquid chromatography (LC) tandem mass spectrometry (MS), coupled with ESI and quantitated using D4-NNN and D4-NNK internal standards.

### KO Mutations in *CLCNt2-S* (*nt2s*) and *CLCNt2-T* (*nt2t*) by Using CRISPR–Cas9 Technology

The CRISPR–Cas9 vector pGREB31 (Addgene) was used for targeted mutagenesis of the *CLCNt2* genes. The backbone of pGREB31 is the CAMBIA vector pCAMBIA1300, which has the *hptII* gene as a selectable marker (hygromycin resistance). After cloning the sequences specifying the desired target site (5′-AGCTGTTGTGAACTATATCG-3′) into the sgRNA cassette of pGREB31, the vector was introduced into *Agrobacterium* strain LBA4404, and T0 plants (TN90) were generated using standard transformation protocols (Horsch et al., 1985). For such KO mutations, we used a TN90 as background, instead of TN90e4e5, to measure the impact of the lack *CLCNt2* copies in a tobacco type used in agronomy, the TN90e4e5 plants being not implemented in the field to date. Nuclease-induced mutations were screened by PCR amplification by using primers flanking the target site, followed by DNA sequence analysis of the amplification products. The primers used for amplifying the *CLCNt2-S* isoform in order to identify CRISPR-induced mutations were Fw 5′-tggagctaaggtctcccacatc-3′ and Rev 5′-ccctgcagcagtaggtgcaaag-3′. The primers used for amplifying the *CLCNt2-T* isoform were Fw 5′-tggagctaaggtctcccatatt-3′ and Rev 5′-ccctgcagcagtaggcgcaaaa-3′.

### Statistical Analysis

Descriptive statistics, such as mean, minimum, maximum, and variance values, were computed per location and per genotype for all compounds investigated in this study. Potential outliers were identified and removed upstream the statistical modeling. One-way fixed effects ANOVA was performed to assess the variation in the different compounds across the different genotypes. The restricted maximum likelihood (REML) objective function was fit with the Newton–Raphson optimizer to obtain the statistical results. For each genotype tested, the genotypic means and the differences between genotypic means (along with their respective CIs) were calculated for each compound. Then, the relative variations of individual compounds were obtained for all comparisons of interest. Relative variation was defined as the effect of a compound concentration in a plant of a given genotype compared with that in a wildtype (WT) plant. Homogeneity of variances across the different genotypes was checked using the Akaike Information Criterion (AIC), and, when necessary, an additional residual variance was added into the linear model with Satterthwaite approximation for degrees of freedom. SAS software 9.4 was used for the statistical treatments, including Procedure MIXED for the computation aspects. R software 4.0.2 and ggplot2 library were used together for producing the charts.

## Results

### *CLCNt2-S* and *CLCNt2-T* Are the Orthologs of *AtCLCa* and *AtCLCb* in *Nicotiana tabacum*

Considering the vacuole as the main storage compartment for nitrate (Martinoia et al., 1981; Granstedt and Huffaker, 1982; van der Leij et al., 1998), and the AtCLCa (De Angeli et al., 2006) and AtCLCb (von der Fecht-Bartenbach et al., 2010) proteins as the tonoplast nitrate/proton antiporters involved in nitrate accumulation, our goal was to measure the impact of silencing or knocking out the tobacco orthologs of *AtCLCa-b* genes on nitrate storage in tobacco leaves. Using tobacco genome resources (Sierro et al., 2014), we were able to confirm that 17 genes encoding CLC proteins are located on the genome, as recently reported by Zhang et al. (2018). Ten NtCLC-like gene products cluster within the subfamily of Cl^−^/H^+^ antiporters, and the remaining seven proteins belong to the subfamily of Cl-channels.

**Figure 2:**
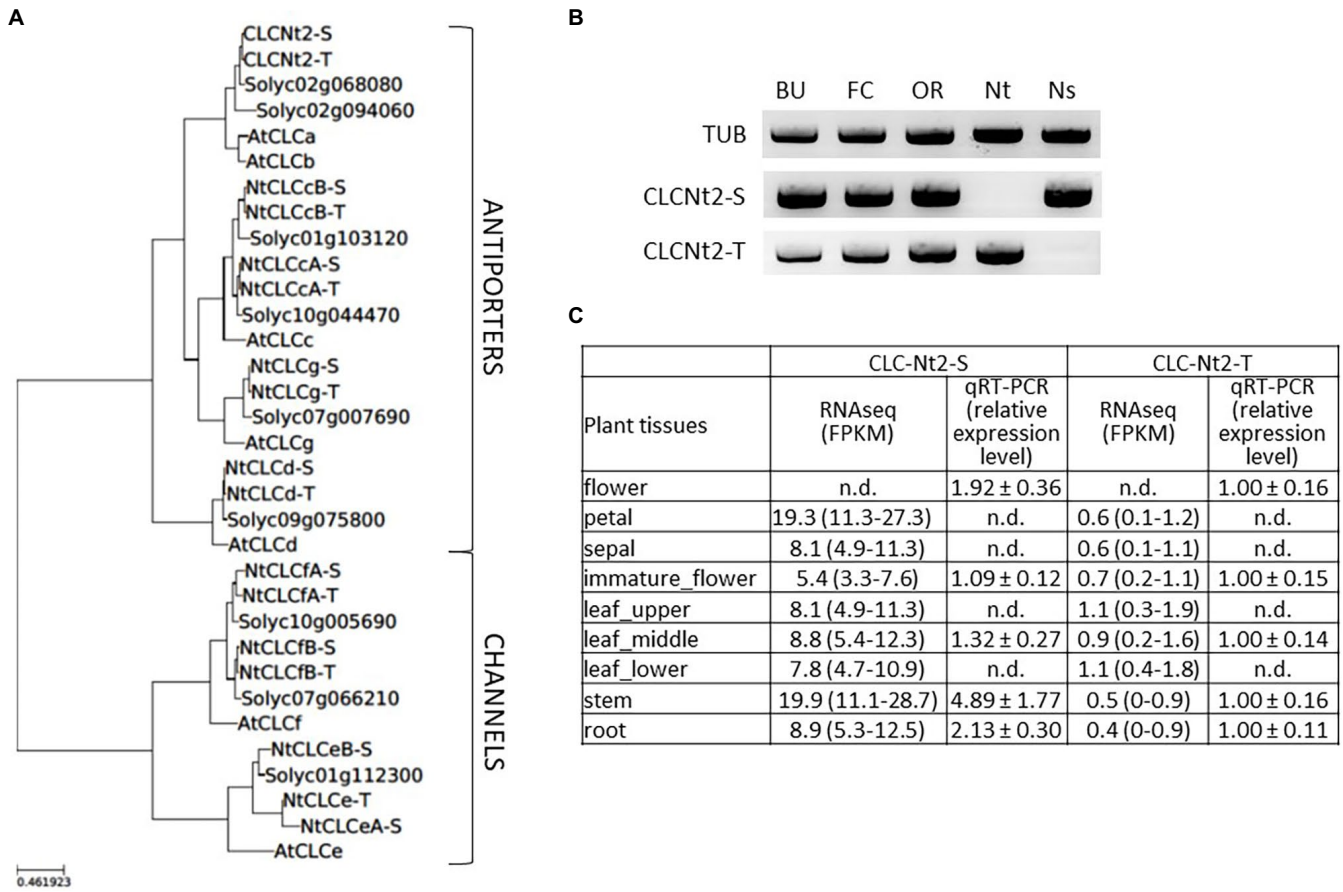
**(A)** Phylogenetic tree of NtCLCs (17 gene products) including the corresponding chloride channel (CLC) orthologs of *Solanum lycopersicum* (nine gene products) and *Arabidopsis thaliana* (seven gene products). The family of NtCLC proteins comprises both chloride/proton antiporters (Cl^−^/H^+^; 10 gene products) and CLCs (Cl^−^; seven gene products). Gene product annotation: AtCLCa (AT5G40890); AtCLCb (AT3G27170); AtCLCc (AT5G49890); AtCLCd (AT5G26240); AtCLCe (AT4G35440); AtCLCf (AT1G55620); AtCLCg (AT5G33280); NtCLCeA-S (Ntab-TN90_AYMY-SS16602:116926-118446); NtCLCeB-S (Ntab-TN90_AYMY-SS16602:130486-145725); NtCLCe-T (Ntab-TN90_AYMY-SS14895:89895-95381); CLCNt2-S (Ntab-TN90_AYMY-SS8473:105857-110645); CLCNt2-T (Ntab-TN90_AYMY-SS15578:189268-193506); NtCLCfB-S (Ntab-TN90_AYMY-SS624: 1786564-1796662); NtCLCfB-T (Ntab-TN90_AYMY-SS9369:96551-105252); NtCLCfA-S (Ntab-TN90_AYMY-SS14737:144497-156283); NtCLCfA-T (Ntab-TN90_AYMY-SS75: 414582-328507); NtCLCcA-S (Ntab-TN90_AYMY-SS925:427910-433271); NtCLCcB-S (Ntab-TN90_AYMY-SS1230:213548-221774); NtCLCcA-T (Ntab-TN90_AYMY-SS16940:1196440-1201435); NtCLCcB-T (Ntab-TN90_AYMY-SS17165:165457-172424); NtCLCd-S (Ntab-TN90_AYMY-SS163770:412-5772); NtCLCd-T (Ntab-TN90_AYMY-SS8957:25454-41730); NtCLCg-S (Ntab-TN90_AYMY-SS1111:2343-8808); and NtCLCg-T (Ntab-TN90_AYMY-SS8106:77848-84317). **(B)** PCR amplification of *CLCNt2-S* and *CLCNt2-T* fragments by using leaf DNA as the matrix from the three main tobacco types—burley (BU), flue-cured (FC), and oriental (OR)—and from the tobacco ancestors *Nicotiana sylvestris* (Ns) and *Nicotiana tomentosiformis* (Nt). **(C)** RNA-seq data showing the expression of *CLCNt2-S* and *CLCNt2-T* copies in tissues of the main organs of mature field-grown BU plants (TN90 background), mean (*n* = 3) calculated with a CI by Cuffdiff2 (version 2.2.1). Relative transcript accumulation of *CLCNt2-S* vs. *CLCNt2-T* in various tissues of greenhouse-grown TN90, according to qRT-PCR analysis (*n* = 3, average and SD).

A phylogenetic tree additionally populated with the seven and nine CLC proteins found in *Arabidopsis thaliana* (Marmagne et al., 2007) and *Solanum lycopersicum*, respectively, is shown in Figure 2A. This allowed us to assign a putative function to the tobacco CLC gene products, principally by sequence analogy subclustering with *A. thaliana* proteins. Eight of the tomato CLCs have duplicated copies in tobacco, one originating from each of the respective ancestors of *N. sylvestris* and *N. tomentosiformis*. The exceptions are Solyc02g094060, which has no homolog in tobacco, and Solyc01g112300, which clusters with the tobacco proteins NtCLCeA-S, NtCLCeB-S, and NtCLCe-T (Figure 2A). Interestingly, the *Arabidopsis* nitrate/proton vacuolar antiporters AtCLCa and AtCLCb (De Angeli et al., 2006; von der Fecht-Bartenbach et al., 2010) also have two orthologous proteins in tomato, Solyc02g0206880 and Solyc02g094060. Only the former isoform, however, appears to have been duplicated in tobacco. CLCNt2-S (*N. sylvestris*-related copy) and CLCNt2-T (*N. tomentosiformis*-related copy) correspond to NtCLC6 and NtCLC1, respectively, in the report by Zhang et al. (2018).

To confirm that the *CLCNt2-S* and *CLCNt2-T* genes are present in all three major commercial tobacco types—BU, FC, and OR—and in the respective *N. sylvestris* and *N. tomentosiformis* tobacco species, we performed PCR amplification of genomic DNA by using specific primers. The data confirm the presence of the *N. sylvestris* and *N. tomentosiformis* copies in the three major commercial types of tobacco (Figure 2B). RNA-seq data of the field-grown BU variety TN90 suggests that the *CLCNt2-S* gene is more highly expressed than *CLCNt2-T* in the main organs of the plant, with the highest expression in the petals and stem (Figure 2C). The results of qRT-PCR assays of the greenhouse-grown TN90 plants also support the conclusion that *CLCNt2-S* is the more highly expressed of the two genes (Figure 2C), despite the differential not appearing as clear as observed with RNA-seq data.

### Suppressed Expression of *CLCNt2-S* and *CLCNt2-T* Affects Nitrate Content and TSNA Production

We used a genetic approach to determine the impact of CLCNt2-S and CLCNt2-T on leaf nitrate content. For this purpose, we generated an RNAi construct to suppress the expression of *CLCNt2-S* and *CLCNt2-T* by incorporating a 407-bp DNA fragment exhibiting 98% identity to both *CLCNt2* sequences. The resulting RNAi constructs were expressed under the control of the constitutive MMV promoter in BU tobacco line TN90e4e5, which harbors KO mutations in CYP82E4 and CYP82E5v2 (Lewis and Dewey, 2015) to prevent excess nornicotine production and, therefore, variable NNN accumulation. To assess the effectiveness of the RNAi suppression, we performed qRT-PCR assays to measure the relative *CLCNt2* transcript accumulation in several T0 individuals and selected the three plants that exhibited the strongest inhibition for further investigation. The cumulative levels of *CLCNt2-S* and *CLCNt2-T* transcripts were decreased by as much as 80% in the *CLCNt2*-RNAi lines (designated CLCNt2-1, CLCNt2-2, and CLCNt2-3) relative to WT controls (Table 1).

**Table 1:** qRT-PCR analyses of the CLC-RNAi lines (CLC2-1, CLC2-2, and CLC2-3) compared with the WT plant.

Genotype	Gene expression (relative expression level)	Biomass (Kg FW/Plant)
WT	0.99 ± 0.01	1.62 ± 0.34
CLCNt2-1	0.21 ± 0.13	1.49 ± 0.41
CLCNt2-2	0.26 ± 0.07	1.59 ± 0.39
CLCNt2-5	0.25 ± 0.12	1.59 ± 0.24

**Figure 3:**
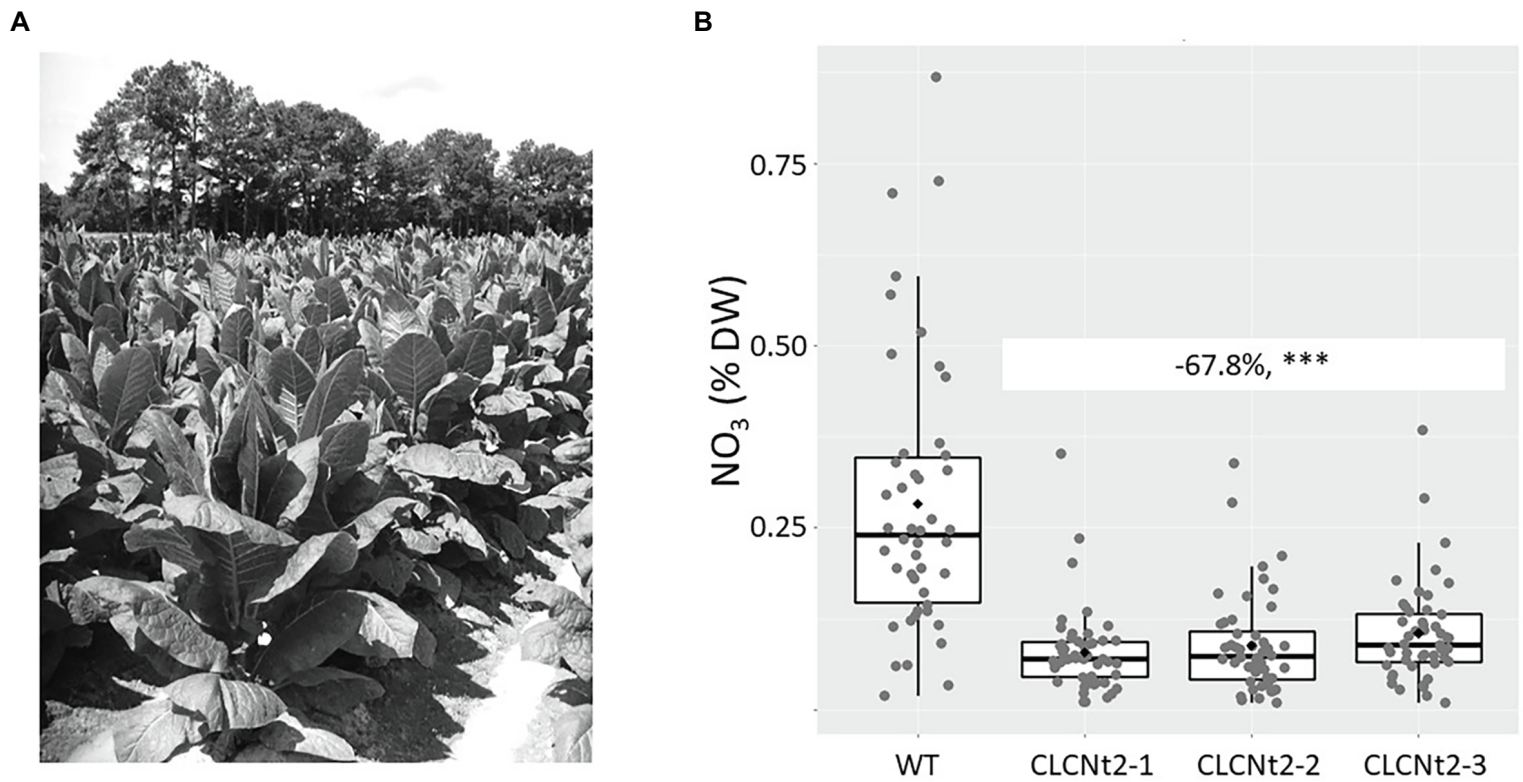
**(A)** Field view of the *CLCNt2*-RNAi (CLC2-1, CLC2-2, and CLC2-3) and wildtype (WT) plant lines grown in a random design. All plant materials of each genotype were pooled from the same field location (between 39 and 47 viable plants per genotype). **(B)** Average nitrate content (%DW) in cured leaf lamina of the WT and CLC-RNAi lines (CLC2-1, CLC2-2, and CLC2-3); boxplots of genotype groups are shown (*n* = 39–50), with the difference of nitrate content observed between the WT and CLCNt2 lines being −67.8% (−51, −84.7%; *p* < 0.0001^***^), as per the results of ANOVA with a CI of 90%.

Average and SD from three analyses (*p* < 0.001); fold changes are expressed as delta–delta ct values (ddCT). Biomass of fresh cut plants at harvest time from field experiment (see Figure 3); average and SD are shown (*n* = 39–50), no statistical differences found.

The *CLCNt2*-RNAi lines and WT control were grown in a field in the United States by using a randomized complete block design. No obvious phenotypic differences were observed between the WT and *CLCNt2*-RNAi lines grown in the field (Figure 3A). Plant biomass—determined by directly weighing plants freshly harvested by cutting at the base of the stalk—was not impacted by the suppressed expression of the *CLCNt2* genes (Table 1). After each plant on the stalk was air-cured for 10 weeks in a dedicated barn in accordance with the standard practice for BU tobaccos,^3^ the lamina of the fourth leaf from the top of each plant was analyzed for nitrate content. In this experiment, the nitrate content in the three independent *CLCNt2*-RNAi lines was reduced significantly (up to 68% lower than the nitrate content in WT plants; Figure 3B).

**Figure 4:**
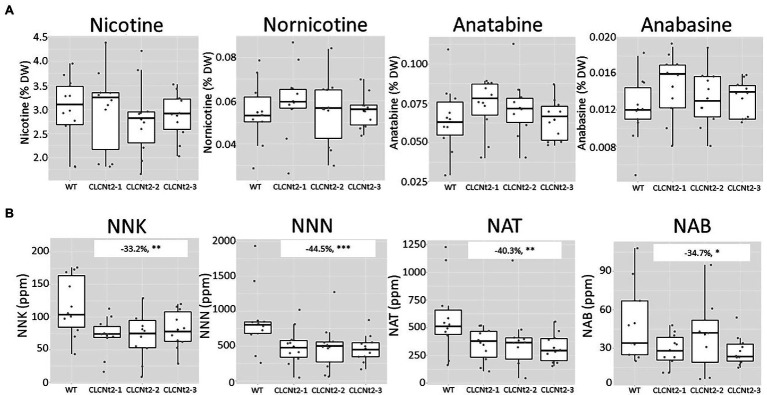
Alkaloid (nicotine, nornicotine, anatabine, and anabasine) and TSNA (NNK, NNN, NAT, and NAB) content in the cured leaf lamina of CLC-RNAi (CLC2-1, CLC2-2, and CLC2-3) and WT plants grown in the field. Data were collected from 10 plants randomly selected after curing. The charts represent the box plots of genotypic groups, with no significant differences in alkaloid content between the WT and CLCNt2 lines **(A)**. For TSNA content **(B)**, the difference observed in NNK levels between the CLCNt2-1 lines and WT was −33.2% (−10.6, −55.8%), *p* = 0.018^**^; for NNN levels, the difference observed between the WT and CLCNt2 lines was −44.5% (−19.8, −69.1%), *p* = 0.004^***^; for NAT levels, the difference observed between the WT and CLCNt2 lines was −40.3% (−14.4, −66.2%), *p* = 0.013^**^; for NAB levels, the difference observed between the WT and CLCNt2 lines resulted in −34.7% (−4.7, −64.6%), *p* = 0.058^*^. An ANOVA test with a CI of 90% was used.

In a randomized subset of the same tobacco samples, we analyzed the impact of decreasing *CLCNt2* expression on alkaloid and TSNA content. Interestingly, the alkaloid content was minimally impacted in the *CLCNt2*-RNAi lines (Figure 4A). In contrast, the TSNA levels in the *CLCNt2*-RNAi lines, individually and cumulatively, exhibited reductions ranging from 33 to 45% relative to the WT. The impact of *CLCNt2* silencing was significant for each TSNA species (NNN, NNK, NAT, and NAB) as well as across the three RNAi lines tested (Figure 4B), which suggested that the nitrosation process is similarly impacted for each of the different alkaloid substrates, nicotine, nornicotine, anatabine, and anabasine.

We determined the TSNAs released in the mainstream smoke of cigarettes made from leaf material from the *CLCNt2*-RNAi and WT plants. The presence of individual TSNAs in mainstream cigarette smoke is primarily due to the thermal release of preexisting TSNAs in the cut filler; although, in some circumstances, pyrosynthesis (which involves TSNA formation in the aerosol or in the hot zone of the cigarette by nitrosation of alkaloids precursors) can additionally contribute to the TSNA yield in cigarette smoke (Jaccard et al., 2018). To investigate the effect of *CLCNt2* suppression on TSNA transfer and/or synthesis in smoke, the cigarettes were made from pure lamina of CLCNt2-1 plants and WT controls collected from the same location over 2 successive years. Cut filler for cigarette production was generated by finely shredding the cured leaf lamina. Differences in environmental growth and curing conditions can lead to substantial differences in TSNA formation, even in tobaccos that share the same genetic background. This phenomenon was evident when the TSNA levels in cigarette smoke were compared between the 2 years (Table 2). For example, twice as much NNN was observed in the smoke from cigarettes produced from materials grown in year 1 than those grown in year 2, even though the smoke nicotine levels were quite similar between the 2 years. The relative differences between the smoke TSNA levels in the *CLCNt2*-*1*-RNAi and WT control; however, were similar across both trial years, with the exception of NAB levels, where the differential was greater in year 2 than in year 1 (Table 2). These data are consistent with the observed reduction in leaf nitrate levels in the transgenic lines, which was also relatively comparable between the two growing seasons: 72% reduction in year 1 and 68% in year 2 for line CLCNt2-1 (data not shown).

**Table 2:** Nicotine, NNN, NNK, NAT, and NAB content in the mainstream smoke of cigarettes made from the lamina of WT and CLC-RNAi line CLC2-1 leaves.

	Year 1				Year 2			
	WT	CLCNt2-1	Percentage	*p*-value	WT	CLCNt2-1	Percentage	*p*-value
Nicotine (mg/cig)	3.17 ± 0.01	3.79 ± 0.15	119%	-	3.15 ± 0.14	2.81 ± 0.07	−89%	-
NNN (ng/cig)	564 ± 12	274 ± 10	−51%	<0.001	269 ± 15	131 ± 4	−51%	<0.001
NNK (ng/cig)	178 ± 13	116 ± 6	−34%	<0.001	144 ± 7	86 ± 1	−40%	<0.001
NAT (ng/cig)	520 ± 32	219 ± 6	−58%	<0.001	349 ± 16	150 ± 6	−57%	<0.001
NAB (ng/cig)	129 ± 12	59 ± 10	−54%	<0.001	65 ± 2	19 ± 1	−71%	<0.001

*For 2 successive years, leaf materials from each genotype were pooled. The data shown represent the combined means and SD from four smoke analyses (*n* = 4). All data are statistically relevant as per pairwise *t*-tests (*p* < 0.001)*.

### *CLCNt2-S* and *CLCNt2-T* KO Impairs Nitrate Storage and TSNA Formation

**Figure 5:**
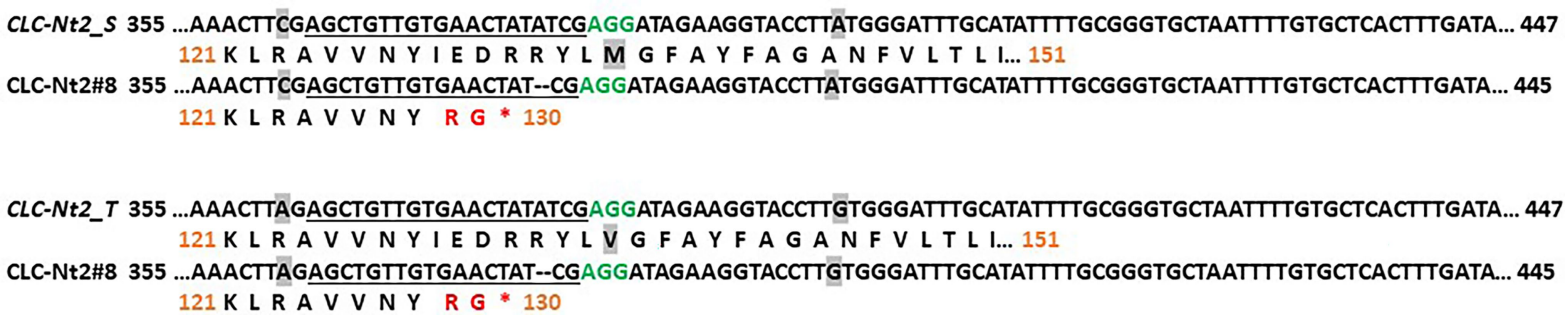
CRISPR–Cas9-induced knockout (KO) mutations in *CLCNt2_S* and *CLCNt2_T*. The region of the WT *CLCNt2* sequence that was targeted by the CRISPR–Cas9 construct is shown on top, with the mutant variants found in line CLCNt2#8 underneath. The 20-bp CRISPR–Cas9 target sequence is underlined, and the PAM site is in green type. Dashes represent bases that have been deleted. WT amino acids are shown in black type; unique amino acids predicted to be present on the C-terminus of the mutant truncated proteins because of frameshifts caused by the two deleted base pairs are shown in red type. Asterisks represent termination codons. Numbers in black correspond to the cDNA sequence beginning at the ATG start codon, and orange numbers indicate the amino acid position. Nucleotides and amino acids that are polymorphic between the *CLCNt2-S* and *CLCNt2-T* isoforms are shaded gray.

Although RNAi suppression is a powerful tool for assessing the consequences of knocking down transcript accumulation of a targeted gene(s), this approach cannot help to establish the effect of complete gene inactivation or assess the relative contribution of multiple genes sharing high sequence identity. To test the effects of complete disruption of *CLCNt2-*S and *CLCNt2-*T gene function, both individually and collectively, we created KO mutations in *CLCNt2* gene copies in the BU background TN90 by using the CRISPR–Cas9 system. In the T0 generation, an individual was identified (designated CLCNt2#8) that was monoallelic for the same 2-bp deletion both at the *CLCNt2-S* and *CLCNt2-T* loci (Figure 5). Given that these small deletions cause a frameshift that would result in the production of highly truncated CLCNt2 proteins that lack over 80% of the normally present amino acids, it can reasonably be assumed that the mutant gene products are incapable of functioning as nitrate transporters.

**Figure 6:**
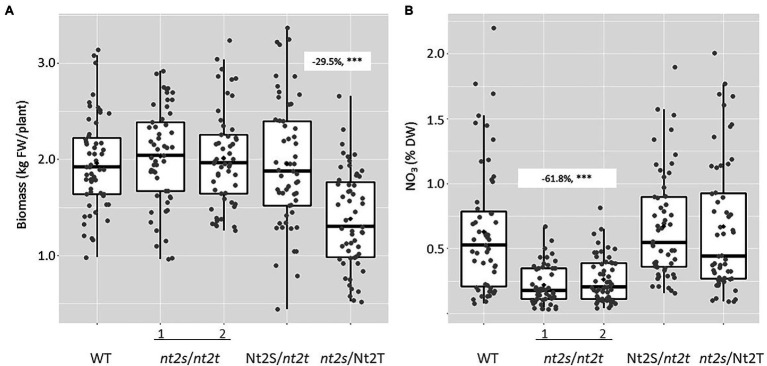
Fresh plant biomass **(A)** and nitrate content **(B)** in the lamina of WT and *clcnt2*-KO mutants. Approximately 50 plants of each genotype (WT, *nt2s*/*nt2t* genotypes 1 and 2, Nt2S/*nt2t*, and *nt2s*/Nt2T) were weighed at harvest. After air-curing, the lamina from the fourth leaf from the top of each plant was assayed for nitrate content. The charts represent boxplots of the five genotypic groups. For biomass **(A)**, the maximum difference observed between the WT and *nt2_s*/Nt2_T plants was −29.5% (−20.9, −38.1%), *p* < 0.001^***^; for nitrate levels **(B)**, the difference observed between WT plants and the two nt2_s/nt2_t lines was −61.8% (−44.6, −78.9%), *p* < 0.001^***^. An ANOVA test with a CI of 90% was used.

Plant CLCNt2#8 was self-pollinated, and over 200 T1 plants were screened by PCR using primers specific for the *nptII* selectable marker gene. Those plants that had lost the CRISPR transgene through segregation were genotyped by DNA sequence analysis in order to select plants homozygous for mutations in the individual *CLCNt2-S* (*nt2s*) and *CLCNt2-T* (*nt2t*) genes (*nt2s*/Nt2T and Nt2S/*nt2t*) as well as the double-homozygous mutant genotype (*nt2s*/*nt2t*). A single line of plants carrying each individual mutation and two double-homozygous mutant lines were grown and evaluated in the field together with the WT as control. After harvest, plant biomass was determined by weighing the plants directly in the field before air-curing, in the same manner as that described above for the *CLCNt2*-RNAi plants. Plant biomass was not affected by the presence of the *clcnt2*-KO mutations in the Nt2S/*nt2t* or double-homozygous mutant lines. The homozygous mutant *clcnt2*_*s* line (*nt2s*/Nt2T), however, unexpectedly displayed a statistically significant 29.5% reduction in biomass relative to the rest of the lines (Figure 6A). After curing, the leaf lamina from the same upper stalk position of each plant was analyzed for nitrate content. Despite the observation that *CLCNt2-S* transcripts were more prevalent than those corresponding to *CLCNt2-T* across every plant tissue examined (Figure 2C), neither single-mutant line displayed a nitrate phenotype that differed significantly from that of the WT control or each other (Figure 6B). In contrast, a significant 61.8% reduction in nitrate was observed in the two double-mutant lines (*nt2s*/*nt2t*), consistent with the data obtained with the *CLCNt2*-RNAi lines (Figure 3B).

**Figure 7:**
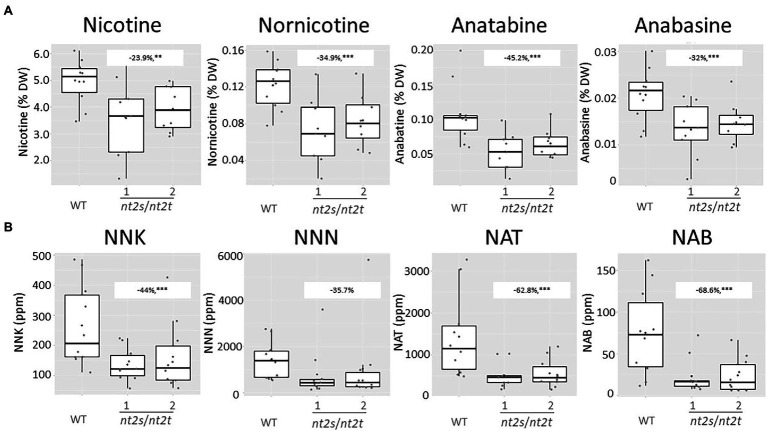
Alkaloid (nicotine, nornicotine, anatabine, and anabasine; **A**) and TSNA (NNK, NNN, NAT, and NAB; **B**) content in the cured leaf lamina of WT and CLC-CRISPR–Cas9 KO double-mutant plants (nt2s/nt2t lines 1 and 2) grown in the field. Data were collected from 10 plants randomly selected after curing. The charts represent boxplots of the genotypic groups. For nicotine levels, the difference observed between the WT and nt2_s/nt2_t lines was −23.9% (−9.1, −38.6%), *p* = 0.010^**^; for nornicotine levels, the difference observed between the WT and nt2_s/nt2_t lines was −34.9% (−17.8, −52%), *p* = 0.002^***^; for anatabine levels, the difference observed between the WT and nt2_s/nt2_t lines was −45.2% (−24.4, −65.9%), *p* = 0.001^***^; for anabasine levels, the difference observed between the WT and nt2_s/nt2_t lines was −32% (−14.1, −50%), *p* < 0.005^***^. In case of the TSNAs, for NNK levels, the difference observed between the WT and nt2_s/nt2_t lines was −44% (−17, −70.9%), *p* = 0.009^***^; for NNN levels, the difference observed between the WT and nt2_s/nt2_t lines was −35.7% (−21.8, −93.2%), *p* = 0.29; for NAT levels, the difference observed between the WT and nt2_s/nt2_t lines was −62.8% (−33.6, −91.9%), *p* = 0.001^***^; for NAB levels, the difference observed between the WT and nt2_s/nt2_t lines was −68.6% (−37.8, −99.4%), *p* = 0.0008^***^. An ANOVA test with a CI of 90% was used.

Alkaloids and TSNAs were measured in the two independent double-KO lines (nt2s/nt2t). Surprisingly, the inactivation of both *CLCNt2* copies in tobacco also significantly impacted the alkaloid content in leaves (Figure 7A), which was not observed when *CLCNt2* transcript accumulation was knocked down in the *CLCNt2*-RNAi lines (see Figure 4A). The decrease was particularly marked for anatabine, which showed a decrease of 45.2% relative to the WT levels. Lower nicotine, nornicotine, and anabasine levels were also found to be statistically relevant in the double-homozygous *clcnt2*-KO plants, with reductions ranging from 23.9 to 34.9% relative to the WT (Figure 7A). As expected, because of the low-nitrate phenotype, the TSNA content in leaves was also substantially affected in the *clcnt2*-KO plants. The levels of all four TSNAs (NNK, NNN, NAT, and NAB) were clearly decreased in the two *clcnt2* double-mutant lines (*nt2s*/*nt2t*), with a reduction of 44% in NNK, 35.7% in NNN, 62.8% in NAT, and 68.6% in NAB levels relative to the WT (Figure 7B). Despite the mean NNN values being lower, this measurement was not deemed significant owing to the exceptionally large variation measured among the plants, as attested by the minimum and maximum percentage values (−21.8 and −93.2%, respectively). Such variations were likely due to the use of TN90 lines as background for developing the *clcnt2*-KO plants, instead of TN90e4e5 in case of the *CLCNt2*-RNAi plant materials. Indeed, the TN90e4e5 cultivar (Lewis and Dewey, 2015) is similar to the Zyvert™ varieties, having lower and more stable nornicotine levels than TN90. Cumulatively, the data show that suppressed expression or inactivation of *CLCNt2* genes resulted in a nitrate-underaccumulation leaf phenotype that impacted the TSNA formation.

## Discussion

We went through the hypothesis that tobacco homologs of *AtCLC2a* and *b* may represent good targets for reducing nitrate, and thus TSNAs. Therefore, to test this hypothesis, we generated RNAi lines to target the *CLCNt2* downregulation. Because the field results of the RNAi lines were promising, this justified going through the effort of knocking-out the two *CLCNt2* gene copies using genome editing to precisely investigate the respective role of S and T homeologous copies of *CLCNt2*.

We have effectively demonstrated that decreasing the expression of *CLCNt2-S* and *CLCNt2-T* in tobacco limits the storage of nitrate in the leaf, thus reducing the propensity for TSNA formation in the cured lamina and in the smoke of cigarettes made from these tobaccos. Mechanistically, this is best explained by a model, whereby less nitrate is available for reduction by nitrate-reducing leaf surface microbes during the curing period, which limits the formation of nitrite and/or other NOx species that can catalyze alkaloid nitrosation.

In a previous study, we demonstrated that constitutive expression of a deregulated NR enzyme dramatically decreases the free nitrate levels in leaves to approximately 5.9% of that observed in the WT material (Lu et al., 2016). The strategy in this case was based on actively increasing the reduction, and thus assimilation, of free nitrate entering the cytosol of the cell. In the present study, inactivation of the two *CLCNt2* copies, either by using a classical RNAi construct or through CRISPR–Cas9-mediated mutagenesis, led to a reduction in free nitrate levels to approximately 30–40% of those observed in WT leaves. On the basis of gene homology with *A. thaliana*, we can reasonably speculate that the CLCNt2 proteins are localized in tonoplast membranes and are responsible for storing nitrate reserves in tobacco leaf vacuoles. The absence of a tonoplast NO_3_^−^/H^+^ exchanger likely prevents vacuolar storage in *CLCNt2*-RNAi or *clcnt2*-KO plants and, therefore, favors the reduction of free nitrate by the endogenous NR toward assimilation. It has been demonstrated that vacuoles from plants grown in the presence of nitrate likely do represent the major nitrate storage organelle in the plant, but not the only one, accounting for about 58% of the total cellular free nitrate content, according to one study (Granstedt and Huffaker, 1982). Stems and, in particular, xylem vessels may also serve as nitrate repositories (Dechorgnat et al., 2011). Our data indirectly suggest that a nitrate storage compartment(s) other than vacuoles could be present in the laminar tissue of tobacco leaves, possibly explaining the lower magnitude of the nitrate reduction in tobacco plants deficient in CLCNt2 function. These plants have normal NR cycles of activation/inactivation compared with tobacco plants that overexpress a permanently activated NR (Lu et al., 2016). Alternatively, a portion of the nitrate remaining in CLCNt2-compromised plants may be due to the presence of additional transporters or NtCLCs whose function has yet to be elucidated. In *Arabidopsis*, two members of the broad-spectrum peptide transporter/nitrate transporter 1 (PTR/NRT1) family have been shown to be localized to the tonoplast, though their substrates could not be defined when expressed in heterologous systems (Weichert, et al., 2012).

Our data confirmed that decreasing tobacco plant free nitrate content has a direct impact on TSNA levels both in cured material and cigarette smoke. In our prior study, field-grown tobacco plants expressing the 35S:S523D-NR transgene exhibited greatly reduced levels of TSNAs in cured material—corresponding to 44, 64, 65, and 32% lower levels of NNN, NNK, NAT, and NAB, respectively, compared with the levels in cured leaf from WT control plants—and thus an observed total TSNA reduction corresponding to 52% (Lu et al., 2016). In the *CLCNt2*-RNAi lines of the present study, we observed similar reductions in NNN and NAB levels in the cured leaf, while the reductions in NNK and NAT levels were less dramatic (Figure 4). Except in the case of NNN, the reductions in TSNA levels in the *clcnt2*-KO lines tended to be greater than those in the *CLCNt2*-RNAi materials. Given the similar nitrate-lowering efficiencies of the two *CLCNt2* inhibition strategies, the enhanced TSNA reduction in the *clcnt2*-KO lines could not be attributed to these plants possessing lower levels of nitrate *per se*. Instead, it is likely that the reduced alkaloid phenotype observed in the double-homozygous *clcnt2*-KO lines accounted for the greater TSNA reduction, as both nitrate and alkaloid contents are highly correlated with TSNA levels (Lu et al., 2021). As nitrate is the fuel for such pathways, secondary metabolite pathways possibly rely more on stored resources than on directly assimilated ones. The absence of a similar trend with NNN is likely because we used the TN90e4e5 genotype that possesses nornicotine-stabilizing mutations in the RNAi-based study, as opposed to the TN90 background we chose for the KO experiments that lack these mutations.

The mainstream smoke from cigarettes made with tobaccos possessing the 35S:S523D-NR construct showed 76% lower total TSNA content than the mainstream smoke from WT cigarettes (Lu et al., 2016); in contrast, the mainstream smoke from *CLCNt2*-RNAi cigarettes showed 52% lower total TSNA levels than WT cigarettes, a value close to that observed in the tobacco filler (48%). This suggests that, in comparison to 35S:S523D-NR cigarettes, cigarettes made of *CLCNt2*-RNAi tobacco generate a more substantial amount of TSNAs by pyrosynthesis during the smoking process (Jaccard et al., 2018), which is consistent with the levels of nitrate measured in the respective tobacco fillers. Indeed, the extremely low nitrate content measured in the 35S:S523D-NR cut filler certainly more actively circumscribes the nitrosation process occurring during pyrosynthesis compared with what one would predict could occur in *CLCNt2*-RNAi cured materials upon combustion, given the greater levels of free nitrate in the latter.

It is necessary to inhibit the function of both of the tobacco *CLCNt2* genes, *CLCNt2-S* and *CLCNt2-T*, to decrease the nitrate levels in WT plant leaf tissues, although *CLCNt2-S* is expressed at substantially higher levels than *CLCNt2-T* in the leaves of TN90 plants (Figure 2C). Consequently, either the level of CLCNt2 protein produced in the cell is greater than that required for storing excess nitrate in the vacuole, or a crosstalk exists between the two genes, where a deficiency in one may lead to upregulation of the other. The biomass of the controls of the CLCNt2-RNAi and clcnt2-KO trials is supposed to be constant. The observed differences capture the effects of different crop seasons and limit the direct comparison of CLCNt2-RNAi and clcnt2-KO plant biomass. Some level of redundancy exists between the two *CLCNt2* genes sharing 98% protein sequence identity, nevertheless, the lower plant biomass of the single *clcnt2-s* compared to control or *clcnt2-t* suggests that the function of the two *CLCNt2* genes may not be totally overlapping (Figure 6A). We have preliminary data suggesting that the flowering time is altered in *nt2s*/Nt2T, but not in Nt2S/*nt2t* plants, in comparison to WT TN90—a phenomenon that could account for differences in plant biomass, as recently documented in our follow-up study on low-nitrate-containing tobacco plants possessing the 35S:S523D-NR gene (Lu et al., 2021). Elucidation of the molecular origin of this phenotype is currently an area of active investigation.

Surprisingly, unlike the results observed when the *CLCNt2*-RNAi knockdown lines were grown in the field, KO of both *CLCNt2* genes resulted in a reduction in the alkaloid content, with anatabine being impacted to the greatest extent. Given that alkaloid production occurs in the root, these results suggest that CLCNt2 activity within this tissue plays a role, whether direct or indirect, in the production of tobacco alkaloids. In most crop fertilization regimens, nitrate is the primary source of nitrogen for biosynthesis of Asp, Orn, and Arg amino acids, from which pyridine amino acids are derived (Dewey and Xie, 2013). One model that could explain these results is that the absence of CLCNt2 function in the root hinders nitrate storage within root vacuoles, leading to a greater proportion of the nitrate being directly mobilized to the aerial tissues of the plant, where it cannot be routed toward alkaloid production. That we did not observe this phenomenon in *CLCNt2*-RNAi plants might be attributable to: (1) the RNAi-mediated suppression of *CLCNt2* being less effective in the root than in the leaf and/or (2) the low levels of CLCNt2 protein still produced in knockdown plants, as opposed to that in KO plants, is sufficient to meet the needs for vacuolar sequestration of nitrate within root tissue. Alternative models could be envisioned whereby the disruption of nitrate storage pools in *clcnt2*-KO plants impacts the production of alkaloids, particularly anatabine, *via* alteration of signaling pathways that regulate alkaloid biosynthesis.

## Conclusion

The plants produced in this study offer new materials for studying the origin of and relationships between nitrogen stocks and the nitrogen pathways leading to alkaloid synthesis, an area of investigation which has yet to be adequately addressed (Xi et al., 2008; Zenkner et al., 2019). From the perspective of practical application, this study provides a promising solution to the problem of TSNA accumulation in tobaccos. Although overexpression of a deregulated NR has also proven effective in decreasing TSNA levels *via* the reduction of cellular nitrate pools (Lu et al., 2016, 2021), that approach is mediated by a transgene and is thus subject to limitations that hinder commercial deployment. Breeding of variants deficient in active *CLCNt2* copies, however, offers a straight path toward the development of new commercial tobacco varieties with reduced TSNA levels. Concerning NNN, the other main carcinogenic TSNA along with NNK (IARC, 2004), a suitable solution for reducing its levels is already available through the Zyvert™ technology, which aims to reduce the levels of its precursor alkaloid nornicotine (Lusso et al., 2017). One could foresee TSNA reduction being maximized in both the tobacco matrix and tobacco smoke through the development of commercial tobacco varieties that combine the CLCNt2-deficiency approach with the Zyvert™ technology.

## Data Availability Statement

The original contributions presented in the study are publicly available. RNA-Seq data can be found here: National Center for Biotechnology Information (NCBI) BioProject database under accession number PRJEB46762.

## Author Contributions

PC, JL, AH, HL, JS, YM, HM, SG, RL, LB, and RD contributed to conception and design of the study. LB and RD organized the database and wrote the first draft of the manuscript. SK performed the statistical analysis. LB, RD, HL, and SK wrote sections of the manuscript. All authors contributed to the article and approved the submitted version.

## Funding

Philip Morris International is the sole source of funding and sponsor of this research.

## Conflict of Interest

LB, PC, AH, SK, HL, JS, and SG were employed by PMI R&D, Philip Morris Products S.A., Neuchatel, Switzerland.

The remaining authors declare that the research was conducted in the absence of any commercial or financial relationships that could be construed as a potential conflict of interest.

## Publisher’s Note

All claims expressed in this article are solely those of the authors and do not necessarily represent those of their affiliated organizations, or those of the publisher, the editors and the reviewers. Any product that may be evaluated in this article, or claim that may be made by its manufacturer, is not guaranteed or endorsed by the publisher.

## Footnotes

^1^ https://www.solgenomics.net/organism/Nicotiana_tabacum/genome

^2^ http://powermatic2.com

^3^ https://www.yumpu.com/en/document/view/11305787/burley-tobacco-production-guide-virginia-tech
